# Backpropagation DNN and Thermokinetic Analysis of the Thermal Devolatilization of Dried Pulverized *Musa sapientum* (Banana) Peel

**DOI:** 10.3390/polym18010122

**Published:** 2025-12-31

**Authors:** Abdulrazak Jinadu Otaru

**Affiliations:** Chemical Engineering Department, College of Engineering, King Faisal University, P.O. Box 380, Al Ahsa 31982, Saudi Arabia; aotaru@kfu.edu.sa

**Keywords:** banana peel, thermochemical conversion, TGA/DTG traces, backpropagation deep learning, thermokinetic

## Abstract

This study examined the thermal degradation of pulverized *Musa sapientum* (banana) peel waste through thermogravimetric measurements and thermokinetic modelling. For the first time, it also incorporated backpropagation deep learning to model pyrolysis traces, enabling the prediction and optimization of the process. Physicochemical characterization confirmed the material’s lignocellulosic composition. TGA was performed between 30 and 950 °C at heating rates of 5, 10, 20, and 40 °C min^−1^, identifying a primary devolatilization range of 190 to 660 °C. The application of a backpropagation machine learning technique to the processed TGA data enabled the estimation of arbitrary constants that accurately captured the characteristic behaviour of the experimental data ($R^{2}\sim 0.99$). This modelling and simulation approach achieved a significant reduction in training loss—decreasing from 35.9 to 0.07—over 47,688 epochs and 1.4 computational hours. Sensitivity analysis identified degradation temperature as the primary parameter influencing the thermochemical conversion of BP biomass. Furthermore, analyzing deconvoluted DTG traces via Criado master plots revealed that the 3D diffusion model (Jander [D3]) is the most suitable reaction model for the hemicellulose, cellulose, and lignin components, followed by the R2 and R3 geometrical contraction models. The estimated overall activation energy values obtained through the Starink (STK) and Friedman (FR) model-free isoconversional kinetic methods were 82.8 ± 3.3 kJ.mol^−1^ and 97.6 ± 3.9 kJ.mol^−1^, respectively. The thermodynamic parameters estimated for the pyrolysis of BP indicate that the formation of activated complexes is endothermic, endergonic, and characterized by reduced disorder, thereby establishing BP as a potential candidate material for bioenergy generation.

## 1. Introduction

The sustainable and efficient utilization of biomass waste through thermochemical conversion into biofuels promotes a more resource-efficient economy by minimizing agricultural waste, extending the lifespan of biomass resources, and encouraging the use of biomass-derived products. Agricultural biomass wastes are naturally organic and renewable [1], which makes their thermochemical conversion a key component of a circular economy that supports sustainable development through solid waste management strategies—reduce, reuse, recycle, and recover (the 4Rs). Biomass-derived materials, such as corncobs and wood pellets, are well recognized as vital feedstocks for biofuels [2]. However, growing demands for renewable energy, coupled with the potential scarcity of traditional pyrolysis feedstocks in the near future, highlight the need to explore the bioenergy potential of other agricultural wastes, particularly banana peels, which are a significant focus of this study.

The thermochemical conversion of various agro-derived wastes has been extensively studied, primarily focusing on estimating the energy required to initiate the process (activation energy) [3] and the thermodynamic parameters necessary for the formation of activated complexes [4]. For example, Dhifallah et al. [3] explored the bioenergy potential of raw *Phragmites australis* (common reed) using a thermogravimetric (TGA) system under an inert nitrogen flow and heating rates ranging from 10 to 50 °C.min^−1^. The overall activation energies estimated in this study using the Flynn-Wall-Ozawa (FWO) and Kissinger-Akahira-Sunose (KAS) model-free isoconversional kinetic methods were 156.98 kJ.mol^−1^ and 158.46 kJ.mol^−1^, respectively. The D3 diffusion model, characterized by the Ginstling-Brownstein equation, was identified as the likely reaction mechanism. Additionally, thermodynamic parameters such as the activation enthalpy (ΔH = 151.24 kJ.mol^−1^), the Gibbs free energy of the activated complex (ΔG = 225.04 kJ.mol^−1^), and the entropy of activation (ΔS = −0.095 kJ.mol^−1^.K^−1^) indicated that the formation of activated complexes is endothermic, non-spontaneous, and more favourable.

Nath et al. [5] studied the thermal decomposition of wheat straw pellets (WSP) using a thermogravimetric analysis (TGA) system in an inert atmosphere. They examined degradation temperatures ranging from 31 to 800 °C and applied three different heating rates: 10, 20, and 30 °C.min^−1^. The findings categorized the TGA traces of WSP decomposition into three phases: drying (moisture removal at 31–161 °C), devolatilization (decomposition of hemicellulose and cellulose from 161–550 °C), and carbonization (decomposition of lignin and extractives from 550–800 °C). This analysis resulted in estimated activation energies of 45.02, 136.04, and 358.11 kJ.mol^−1^ for each respective phase. Otaru and Albin Zaid [6] reported activation energy (E_A_) values of 84.09 kJ.mol^−1^, activation enthalpy (ΔH) of 79.19 kJ.mol^−1^, and Gibbs free energy of the activated complex (ΔG) of 259.30 kJ.mol^−1^ for the thermal decomposition of palm fronds, indicating its potential as a suitable pyrolysis feedstock for bioenergy generation. Additionally, they found a positive activation entropy value of 0.308 kJ.mol^−1^.K^−1^, suggesting a monomolecular reaction [7,8] associated with the thermal decomposition of palm fronds. Furthermore, various other agro-derived wastes, such as *Typha latifolia* [9], coffee husk [10], groundnut shell [10], and soybean straw [11], have also been investigated for their bioenergy potential.

Numerous research studies have investigated the sustainable utilization of banana peel waste, with most focusing on its physicochemical characterization and thermal analysis to assess its potential for biofuels. For example, Chua et al. [12] found that banana peel contains 76.47% volatile matter based on experimental TGA analysis, indicating its viability as a pyrolysis feedstock. Their study estimated the bio-oil heating value at 38.88 MJ/kg at 300 °C, with bio-oil, biochar, and syngas yields from the pyrolysis process calculated at 16.60%, 36.45%, and 46.96%, respectively. Kumar et al. [13] employed the FWO, KAS, Friedman (FR), and Coats-Redfern (CR) kinetic methods to estimate the activation energy for the thermal decomposition of banana peel in a specially designed fixed-bed reactor. The activation energies ranged from 108.42 to 201 kJ.mol^−1^, with significant bio-oil yields of 11.47, 12.50, and 12.88 mL/100 g of biomass at pyrolysis heating rates of 5, 10, and 15 °C.min^−1^, respectively. Ameha et al. [14] conducted a thorough physicochemical and thermogravimetric (TGA) analysis of banana peel waste, revealing a devolatilization temperature range of 350 to 500 °C for the thermal degradation of hemicellulose and cellulose components. The breakdown of the lignin composition in biomass material into smaller components, such as carbon dioxide, was reported to show a steep decline, typically occurring above 600 °C in TGA traces. Mishra et al. [15] reported that the vaporization of adsorbed moisture in the lignocellulosic fibre during banana peel pyrolysis occurs at 100 °C, while the decomposition of glycosyl units in hemicellulose and cellulose takes place within the temperature range of 278 to 440 °C.

Despite numerous studies on the thermochemical conversion of banana peel, the existing literature on the thermokinetic analysis of TGA traces derived from the pyrolysis of this biomass material remains limited. Recent advancements in modelling and simulation techniques utilizing deep learning tools have been employed to predict TGA traces and optimize experimental operating parameters for biomass pyrolysis [16,17,18]. However, to the best of the author’s knowledge, there is a paucity of studies that apply deep neural network (DNN) analysis to TGA traces specifically from banana peel pyrolysis. This study presents, for the first time, a comprehensive analysis of TGA traces resulting from the thermochemical conversion of pulverized *Musa sapientum* (banana) peel, employing machine learning backpropagation and thermokinetic assessments. The determination of reaction modelling, thermokinetic data, and the application of DNN to the thermochemical conversion of banana peel are essential for evaluating the energy balance and sustainability of the process, as well as for identifying the specific parameters that maximize the yields of bio-oil, biochar, and syngas. The selection of backpropagation machine learning modelling and simulation techniques or DNN in this study, as opposed to traditional supervised machine learning models such as Support Vector Machines (SVM), Regression Models, K-Nearest Neighbours (KNN), Naïve Bayes, Decision Tree and Ensemble Modelling techniques is justified by its superior capability to model non-linear relationships and effectively capture intricate patterns [19]. Furthermore, the raw TGA traces collected at varying heating rates often result in extensive non-linear datasets; a well-trained DNN model is adept at managing such large datasets, thereby facilitating the extraction of meaningful features, learning nuanced patterns, and enhancing interpretability [20]. The findings are anticipated to contribute to a circular economy approach that promotes the sustainable and efficient utilization of solid waste, in alignment with the Saudi Green Initiative, the European Union’s Strategic Plan on Waste Management, and the United Nations Sustainable Development Goals 1, 3, and 13.

## 2. Experimental Procedure

The experimental procedure in this study involved three main steps: pulverizing dry banana peel, characterizing the pulverized sample, and conducting thermogravimetric (TGA) analysis on the sample. The banana peel used in this research was sourced from a local shop located in Al Ahsa Governorate in the Kingdom of Saudi Arabia. Initially, the peel was air-dried for 12 days to remove excess moisture. It was then pulverized into a fine powder with particle sizes of ≤300 µm. This size range was chosen to minimize the influence of thermal contact surfaces associated with larger particles, as noted in reference [21], which indicated that this range is optimal for evaluating functional and physicochemical properties. The apparent (bulk) density of the pulverized banana peel waste was measured at 0.42 g.cm^−3^, while the compressed density was measured at 0.46 g.cm^−3^. Following these measurements, elemental analysis, morphological characterization, and functional group analysis of the pulverized samples were conducted prior to the thermogravimetric analysis. The details of these analyses are described as follows:

### 2.1. Proximate and Ultimate Analysis

The proximate analysis of the pulverized sample was performed to determine its moisture, volatile matter, fixed carbon, and ash contents, following a procedure similar to that described in [22]. A Perkin-Elmer thermogravimetric analyzer was used for this analysis. To measure moisture content, the sample was heated from 30 to 107 °C at a rate of 10 °C.min^−1^ for one hour in an inert nitrogen atmosphere until a constant weight was achieved. The weight loss recorded during this process indicated the moisture content. The volatile matter content was evaluated by heating the sample to 600 °C for 8 min in the same inert nitrogen atmosphere. Finally, to ascertain the ash content, the sample was heated to 900 °C for 90 min until it reached a constant weight.

The ultimate analysis of the pulverized agricultural waste was performed to determine the carbon, hydrogen, nitrogen, sulphur, and oxygen content in the sample (i.e., the CHNS-O compositions [23]). A 2.2 mg sample was weighed, tightly wrapped in an aluminum foil crucible, and placed in a VELP Scientifica EMA 502 Elemental Micro Analyzer (Usmate, Italy). The sample was preheated in the combustion chamber using helium gas at a flow rate of 120 mL.min^−1^. Oxygen gas was then introduced at 300 mL.min^−1^ to convert the elements into their gaseous forms. The resulting gases were passed through a thermal conductivity detector (TCD) operating at 60 °C, which measured the CHNS-O compositions, with the data recorded by the VELP Standard EMA Soft™ data acquisition software.

### 2.2. SEM/EDS and FTIR Measurements

Scanning electron microscopy (SEM) and energy-dispersive X-ray spectroscopy (EDS) measurements were conducted on the sample using a JCM-700 NeoScope SEM fitted with a backscattered electron detector. A pulverized banana peel sample was placed on a stub and coated with a gold-palladium alloy to enhance image clarity from the SEM system [24]. The SEM analysis of the coated sample was performed at an accelerating voltage of 15 kV and a working distance of 8 to 15 mm. EDS measurements were also taken by capturing images and assessing elemental compositions at various locations within the coated sample. Additionally, Fourier transform infrared (FTIR) measurements were carried out on the pulverized sample to identify molecular compounds. This analysis utilized an Agilent Cary 630 FTIR analyzer with version 5.8 of Agilent MicroLab software, following a procedure described in [25]. The transmittance of infrared radiation for the sample was measured across wavelengths of 4000 to 400 cm^−1^ to identify various absorption peaks, which were compared to an established FTIR database referenced in [26]. To ensure accurate measurement of infrared transmittance, the crystal section of the FTIR system was cleaned with acetone before sample placement, and a pressing mechanism was used to securely hold the sample against the crystal section during measurement.

### 2.3. TGA/DTG Measurements

Thermogravimetric analysis (TGA) of pulverized banana peel (BP) was conducted using a Perkin Elmer TGA 4000 analyzer equipped with Pyris Series data acquisition software. This analysis aimed to evaluate the thermal degradation (weight loss) of the sample over a specified temperature range, following methodologies like those reported in [27,28,29,30]. Initially, the aluminum crucible (sample holder) was cleaned and placed in the combustion chamber of the TGA analyzer, where the sample weight was calibrated to zero. A sample was placed in the TGA’s crucible, ensuring it completely covered the bottom. This helps enhance heat transfer and minimize thermal gradients within the sample. To maintain an inert environment for pyrolysis, nitrogen gas was introduced into the TGA combustion chamber at a flow rate of 20 mL.min^−1^. The Pyris software (Pyris TGA 8000) was configured for a heating rate of 20 °C.min^−1^ and a degradation temperature range of 30 to 950 °C to regulate the TGA operating conditions. Weight loss data were systematically recorded in relation to the degradation temperature, with the duration of the analysis primarily influenced by the heating rate. Following each experimental run, the thermogravimetric analyzer was allowed to cool for 30 min to room temperature before subsequent experiments were conducted at heating rates of 5, 10, and 40 °C.min^−1^. To ensure the reproducibility and repeatability of the data, the thermogravimetric analysis was conducted twice more across all heating rates and varying temperature conditions. The results indicated differences within a ±4 percent deviation, with R-squared values ranging from 0.992 to 0.998 when compared to the initial experimental measurements for each heating rate. The thermogravimetric analysis (TGA) data obtained for these biomass samples were subsequently utilized to estimate the derivative thermogravimetric (DTG) data by dividing the changes in weight loss (mg) by the differences in temperature across all four heating rates and the entire pyrolysis temperature range.

## 3. Analysis of Experimental Data

### 3.1. Compositional Analysis

The proximate and ultimate analyses data obtained for the pulverized banana peel (BP) are presented in Table 1a and Table 1b, respectively. Table 1a shows that the moisture content is 6.5%, while the fixed carbon, volatile matter, and ash contents are 18.7%, 67.8%, and 7.0%, respectively. The presence of a high amount of volatile matter suggests that the BP is a viable feedstock for pyrolysis operations, which could lead to a significant amount of devolatilization of the material during thermolysis [31,32,33]. These proximate analysis data are similar to those reported in [33] for raw banana peel sourced from Indonesia, yielding values of 6.1%, 16.7%, 68.2%, and 9.1% for moisture, fixed carbon, volatile matter, and ash contents, respectively. In another related study, Khansaw et al. [34] estimated moisture and ash contents of 4.9% and 8.2%, respectively, for Nam Wa Mali-Ong banana peel sourced from Thailand. Ultimate analysis data presented in Table 1b showed high carbon (39.7%) and oxygen (52.3%) content, with minor amounts of hydrogen (4.9%) and nitrogen (3.1%). Kabenge et al. [32] reported high values of 35.7% for carbon and 46.0% for oxygen in raw BP biomass sourced from Makerere University, Kampala, Uganda. They also found minor amounts of hydrogen (6.2%) and nitrogen (1.9%). The elevated levels of carbon and oxygen indicate the lignocellulosic nature of the BP biomass [35], which was characterized [32] as containing 41.3% hemicellulose, 9.9% cellulose, and 8.9% lignin. In contrast, the low nitrogen content and absence of sulphur suggest that the thermochemical conversion of BP biomass waste is environmentally friendly, as it may result in lower emissions of harmful gases such as NOx and SOx [13].

### 3.2. SEM/EDS and FTIR Data

**Figure 1:**
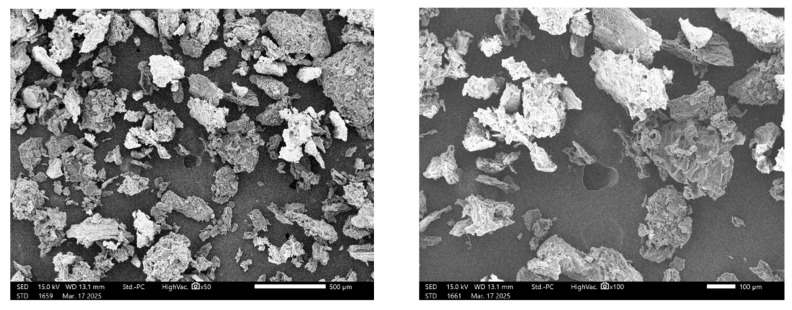
Scanning electron microscope (SEM) images of pulverized banana peel.

Figure 1 displays scanning electron microscope (SEM) images of pulverized raw BP biomass, while Table 2 provides energy dispersive X-ray spectroscopy (EDS) data detailing the elemental composition of the material. The SEM micrographs reveal irregular structures with low sphericity (sub-angular, angular, and very angular) and characteristic particle dimensions ranging from 100 to 300 µm. Additionally, the micrographs indicate porous surfaces within the particles, which may enhance their water retention capacity on a wet basis [36]. Table 2 highlights a significant presence of carbon (39.7%) and oxygen (37.2%) in the pulverized BP biomass, aligning with the ultimate analysis data presented in Table 1b. The presence of gold (Au) at 3.4% is due to the gold-palladium coating applied to the pulverized BP biomass material before SEM/EDS analysis. Furthermore, potassium (15.9%) and chlorine (3.7%) contribute to the high ash content of the BP biomass, as indicated by the proximate analysis data in Table 1a.

**Figure 2:**
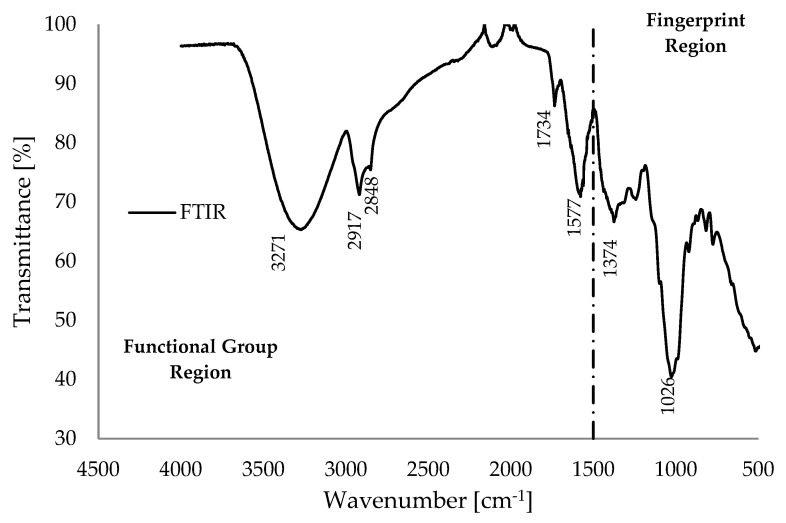
Fourier transform infrared spectroscopy (FTIR) measurements of the pulverized banana peel.

Figure 2 displays the Fourier transform infrared (FTIR) spectrum of pulverized BP biomass material, highlighting several absorption peaks categorized into functional groups (above 1500 cm^−1^) and the fingerprint region (below 1500 cm^−1^). The functional group region is useful for molecular identification, while the fingerprint region is generally considered less reliable for this purpose [37]. Table 3 outlines the FTIR absorption peaks, referencing data from the FTIR database in [26]. For example, the broad peak at 3271 cm^−1^ indicates O-H stretching associated with alcohol in the hemicellulose and cellulose composition, while the absorption peaks at 2848 cm^−1^ and 2917 cm^−1^ suggest C-H stretching of alkanes. Memon et al. [38] reported similar FTIR spectra for BP biomass, with characteristic peaks at 3313 cm^−1^, 2920 cm^−1^, and 2851 cm^−1^, which indicate the presence of alcohol, alkane, and carboxylic acid or ester, respectively. The sharp peak 1577 cm^−1^ corresponds to C-H stretching of aldehydes and C=C stretching of the aromatic ring found in lignin [26]. The pronounced peak observed at 1734 cm^−1^ is attributed to the carbonyl vibration band of acetyl groups (C=O) associated with the ester functional group in pectin [39]. Furthermore, the peaks at 1374 cm^−1^ and 1026 cm^−1^ likely correspond to O-H bending in alcohols and C-O stretching vibrations within the primary biopolymers (hemicellulose, cellulose, and lignin) of the BP biomass, respectively [40]. Overall, the Fourier-transform infrared (FTIR) spectrum of banana peel (BP), as depicted in Figure 2, exhibits characteristic peaks that are consistent with the hydroxyl and carbohydrate regions. Notably, the FTIR spectrum of the pulverized BP biomass demonstrates a higher pectin content, as evidenced by the prominent peak at 1734 cm^−1^ in Figure 2, compared to many other lignocellulosic materials [41]. Additionally, the FTIR spectra of banana peel biomass display slight variations when compared to those of other lignocellulosic biomass, which can be attributed to the presence of a broadened hydroxyl band in the FTIR spectra presented in Figure 2. This observation suggests interactions between metal ions and functional groups, facilitated by the significant presence of potassium (as indicated in Table 2) within the materials [41,42].

### 3.3. TGA/DTG Data

**Figure 3:**
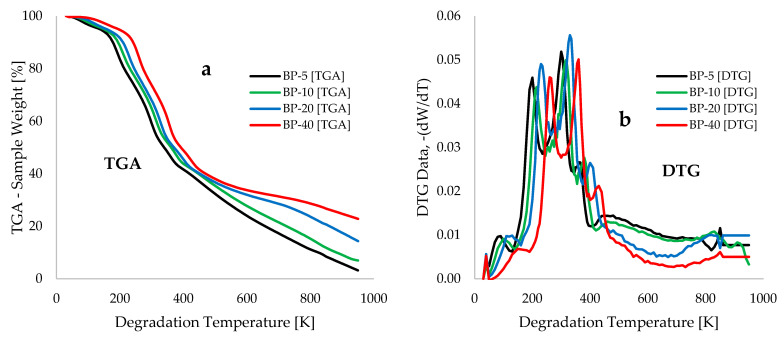
Plots of experimentally measured data showing (**a**) TGA—sample weight [%], and (**b**) DTG—(dW/dT) against degradation temperature [K].

Figure 3 displays the TGA and DTG traces for the thermal degradation of raw BP biomass waste at heating rates of 5, 10, 20, and 40 °C.min^−1^, with degradation temperatures ranging from 30 to 950 °C. In Figure 3a, the TGA traces shift to higher temperature maxima as the heating rates increase. For example, at a 10 wt% loss, the temperature values are 178.2, 194.2, 209.6, and 241.6 °C for the heating rates of 5, 10, 20, and 40 °C.min^−1^, respectively. At a 70 wt% loss during pyrolysis, the corresponding high temperature values are 525.3, 567.8, 655.9, and 754.9 °C for these heating rates. This observed shift in the TGA traces aligns with findings reported in [13] regarding the thermal decomposition of dry banana peel at heating rates of 5, 10, and 15 °C.min^−1^. Figure 3 illustrates that the weight losses during the pyrolysis of BP biomass can be divided into three stages: drying, devolatilization, and carbonization. The DTG traces in Figure 3b indicate temperature peaks of 100 °C and 160 °C for BP degradation at heating rates of 5 °C.min^−1^ and 40 °C.min^−1^, respectively, which correspond to the loss of moisture in the biomass. During the drying stage, a temperature range of 100 °C to 190 °C was observed, leading to weight losses between 5.8% and 8.7%. Ameha et al. [14] estimated a moisture loss of 9.2% during the pyrolysis of banana peel, while another study reported a moisture loss of 8.6% occurring between 100 °C and 250 °C [13]. Although the SEM micrograph in Figure 1 clearly shows the porous structure of BP biomass, which enhances its water retention capacity when wet and its water release capacity when dry, the moisture content of BP (6.51%) listed in Table 1a is below the maximum acceptable limit of 10% for biomass pyrolysis operations [13].

Figure 3b illustrates the devolatilization or pyrolysis stage of BP thermal degradation, which occurs within the temperature range of 190–660 °C and results in a 62.9 wt% loss. This stage is marked by a significant release of both volatile and non-volatile gases, including hydrogen, carbon monoxide, carbon dioxide, and methane [13]. Three distinct DTG peak ranges were identified across the heating rates considered in this study (5–40 °C·min^−1^): 200–255 °C, 320–355 °C, and 380–440 °C. The first two peak ranges correspond to the decomposition of hemicellulose and cellulose fibres in the BP biomass, while the peak range of 380–440 °C is associated with the higher thermal resistance of lignin within the BP biomass [15]. The multiple DTG peaks observed in Figure 3 can be attributed to the presence of potassium (an alkali metal) in the materials, as indicated by the energy dispersive spectroscopy (EDS) data presented in Table 2. The significant concentration of this alkali metal serves as a catalyst during the thermal devolatilization of the pulverized biomass material, thereby altering the decomposition pathways and resulting in multiple and complex DTG patterns [42,43,44]. This contrasts with biomass materials characterized by lower concentrations of alkali and alkaline earth metals, such as pinewood, bagasse, and various pretreated agricultural residues [45,46]. Above 660 °C, the carbonization stage of BP degradation is evident in the TGA and DTG traces shown in Figure 3, marked by the gradual degradation of lignin and extractive components. This slow degradation is attributed to the breakdown of lignin into smaller components, such as carbon dioxide [14], leading to a steep decline towards the char region [13]. Figure 3b indicates that devolatilization accounts for the highest weight loss among the three stages. This finding aligns with previous research [47,48], which indicates that hemicellulose and cellulose are hydrolysed by extracellular enzymes, enhancing their susceptibility to water and facilitating their earlier degradation compared to lignin, which is characterized by complex bonds and cross-linkages.

## 4. Backpropagation Deep Learning Approach

This study adopted the backpropagation deep learning approach to determine and optimize the arbitrary constants (synaptic weights and biases) essential for accurately predicting TGA traces within a deep neural framework. It also aimed to understand the parametric influences on the characteristic behaviour of the pyrolytic process. To achieve this, a deep neural network (DNN) framework with two hidden layers and consisting of two neurons each (DNN [2,2]) was first created. The training of experimentally measured data was then performed using mathematical differential equations and learning algorithms referenced in [45,46]. Previous reports by this group in [47,48] utilized general artificial neural network models (Equations (1)–(3)) [49], and implemented the gradient descent algorithm, which operates from output to input (i.e., the backpropagation method). This method formulated learning algorithms capable of training the experimental data in the direction of the negative gradient [50], with adjustments in the learning rate or step size [51] until a global minimum was reached.

(1) $$Z=\sum_{i}w_{i}.x_{i}+b_{k}$$

(2) $$a=\sigma \prime [z]=\frac{1}{1+e^{-z}}$$

(3) $$C={\left(y-a\right)}^{2}$$

where Z is the sum weight, $w_{i}$ is the synaptic weight, $b_{k}$ is the bias, $x_{i}$ is the input function, a is the activation function, σ′[z] is the logistic function of z, C is the training loss or cost function, and y is the experimentally measured output function.

The application of learning algorithms to train TGA traces necessitates a clear understanding of the experimentally measured datasets, which must be categorized into inputs (features) and outputs (labels), followed by data-pruning. In this study, the weight loss of the BP sample during thermochemical conversion is identified as the output parameter, while the heating rates, degradation temperature, and degradation time serve as input parameters. Data-pruning involved selecting key experimental data points from a total of 21,415 raw measurements for each variable. Specifically, 448 data points were selected for each of the four heating rates, using a temperature spacing of 10 °C during the drying (moisture loss) and carbonization stages. In contrast, a finer temperature spacing of 5 °C was employed during the critical degradation stage (devolatilization) of the pyrolytic process, which occurs at degradation temperatures between 190 and 660 °C. This data-pruning process in machine learning enhances efficiency by reducing the computational time and resources needed for training. This efficiency is described as Kolmogorov complexity, which represents the shortest computer programme capable of generating a specified output in a reduced amount of time [52].

Log-normalization of the selected experimental data points was performed to facilitate the application of the Sigmoid activation function (Equation (2)) in predicting output signals within the range of 0 to 1 [53]. This process involved dividing the selected data by their corresponding maximum realistic values. For instance, the heating rate was divided by a maximum value of 100 °C·min^−1^, while the degradation temperature and degradation time were divided by values of 1000 °C and 11,215 s, respectively. The training of this data was conducted using Microsoft Excel, augmented by a Visual Basic for Applications (VBA) script to update the arbitrary constants in the direction of the negative gradients. The initial time, arbitrary constants, and learning rate were set to 1 s, 0.1, and 5.0, respectively, and these parameters were subsequently updated throughout the training process until the desired local minimum was achieved.

**Figure 4:**
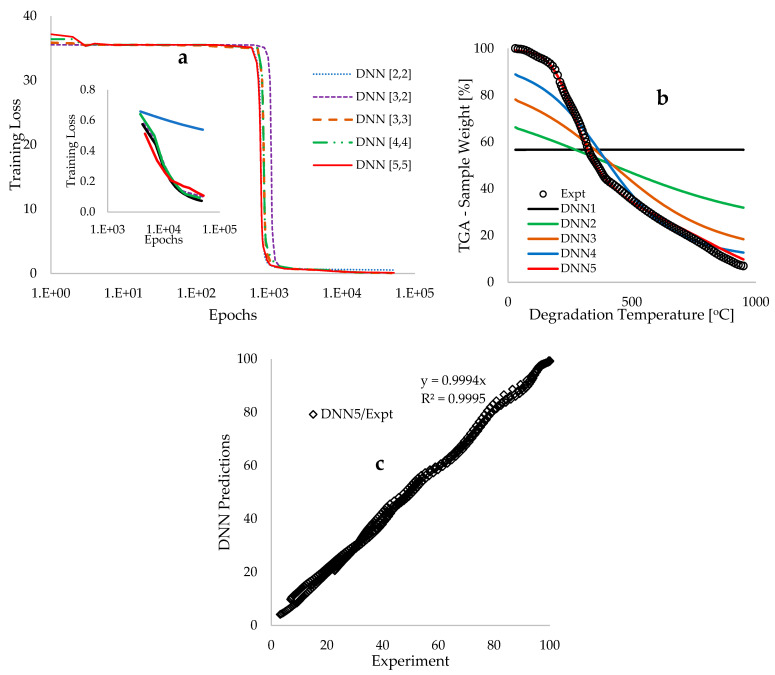
Graphical representations of (**a**) DNN training loss plotted against epochs, (**b**) the comparison of predicted and measured TGA sample weight loss [%] against degradation temperature [°C] at a heating rate of 10 °C.min^−1^, and (**c**) the DNN predictions in relation to the measured TGA weight loss across all heating rates.

Figure 4a illustrates the plots of training losses as a function of epochs (number of iterations) derived from the modelling and simulation of BP biomass pyrolytic data across various deep neural network (DNN) architectures. The training commenced with an initial DNN architecture characterized by two hidden layers, each comprising two neurons, referred to as the DNN [2,2] framework. As depicted in Figure 4a, the computational training loss associated with this DNN framework decreased from 35.54 to 0.54, utilizing 50,053 data points over a period of 1.50 h. It is important to note that an initial training time of 1 s was employed for the computation of each epoch; however, this was subsequently adjusted to zero to expedite the training process, thereby reducing the overall training duration. Additionally, model regularization was implemented through a systematic adjustment of the learning rate from 5 to 3, which proved effective in preventing the occurrence of exploding gradients [54] and ensuring that the computation of the gradient descent equation in the direction of negative gradients did not become trapped in local minima [51].

The final training loss of 0.54 achieved for the DNN [2,2] framework corresponds to a true error of 1.52%. Subsequent modelling modifications were implemented through a systematic increase in the number of hidden neurons (hyperparameter tuning), as the initial configuration of four hidden neurons was deemed relatively low. The objective of this hyperparameter tuning was to further minimize the training loss to reach a global minimum, thereby ensuring that the resulting model captures a more generalized pattern (to avoid overfitting) rather than merely fitting the experimental data (to avoid underfitting) [47]. The systematic addition of hidden neurons, followed by modifications and training, culminated in an optimal architecture comprising two hidden layers, each containing three hidden neurons, referred to as the DNN [3,3] framework. This approach successfully reduced the training loss from 35.88 to 0.08, utilizing a reduced number of epochs totaling 47,688 and computed in 1.37 h. As illustrated in Figure 4a, further increases in the number of hidden neurons do not significantly affect the reduction in the training loss below the 0.07 achieved with the DNN [3,3] architecture; thus, this reduced training loss value is considered the global minimum.

Figure 4 illustrates a systematic improvement in the modelling outcomes of the Thermogravimetric Analysis (TGA) weight loss across various degradation temperatures. The initial modelling outcome (DNN1), derived from arbitrary initial guesses of 0.1, resulted in a constant TGA sample weight of 56.69 wt% over the degradation temperature range of 30 to 950 °C. However, subsequent training and parameter updates in the direction of the negative gradient led to enhancements in the output signals, culminating in a convergence of the predicted Deep Neural Network (DNN) model with the experimentally measured data. This convergence is characterized as the position of minimally achievable training loss, referred to as the global minimum [51]. Figure 4b demonstrates a strong correlation between the modelled and experimental measurements, yielding a coefficient of determination (R^2^) of 0.9995, thereby reinforcing the reliability of the trained arbitrary constants. Additionally, Table 4 presents a statistical comparison of the DNN modelled and experimental data, indicating estimation uncertainties $\left(∆\right)$ of 1.36 and 1.37 wt%, respectively, alongside a mean bias error of 0.02.

**Figure 5:**
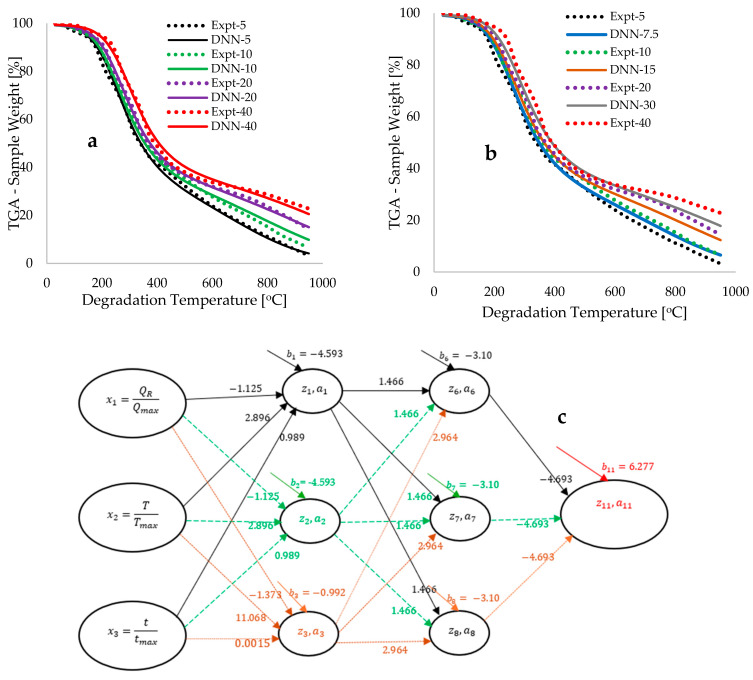
Graphical representations of experimentally measured data alongside (**a**) DNN predicted outcomes, (**b**) interpolated DNN data against degradation temperature [°C], and (**c**) an optimized DNN framework featuring six hidden neurons organized in two layers, interconnected through synaptic weights and biases.

Figure 5a illustrates plots of TGA sample weight against degradation temperature, demonstrating complete overlaps between modelled and experimental measurements for heating rates of 5, 10, 20, and 40 °C·min^−1^. This finding suggests that the model effectively learned to predict the experimental data utilized. Additionally, Figure 4b presents interpolated TGA traces at heating rates of 7.5, 15, and 30 °C·min^−1^, which fall within the expected experimental variability. This observation indicates that the trained model has also acquired generalized patterns of predictions that align with the operational conditions and variability of the experiments. Figure 5c showcases the optimized DNN framework, characterized by two hidden layers, each containing three hidden neurons interconnected by twenty-one synaptic weights ($w_{i}$) and six biases ($b_{k}$). The estimated values of the biases provide an offset in the activation function (output) during training, while the synaptic weight values signify the importance of the input activation on the resulting output signal. Consequently, the computed synaptic weight values in Figure 5c indicate that degradation temperature is the most sensitive feature (input parameter) influencing the thermochemical conversion of BP biomass waste, followed by degradation time and heating rate.

## 5. Thermokinetic Analysis

### 5.1. Thermokinetic Equations

A comprehensive kinetic and thermodynamic analysis of the thermochemical conversion of pulverized BP biomass material was performed to ascertain both the kinetic triplet and thermodynamic parameters. The kinetic triplet parameters include the reaction mechanism, activation energy ($E_{A}$), and pre-exponential factor (A). The thermodynamic parameters requisite for the formation of activated complexes comprise activation enthalpy (∆H), entropy of activation (∆S), and Gibbs free energy of the activated complex (∆G). The Criado master plots, in conjunction with sixteen selected solid-state reaction mechanisms, were employed to estimate the potential solid-state reaction mechanisms for the process. Equation (S1) (Supplementary File) delineates the mathematical expression for the Criado master plots, illustrating the parameters for the estimation of the reduced experimental $\left\{{\left(\frac{T_{x}}{T_{0.5}}\right)}^{2}\frac{{\left(dx/dt\right)}_{x}}{{\left(dx/dt\right)}_{0.5}}\right\}$ and theoretical $\left\{\frac{f\left(x_{i}\right).g\left(x_{i}\right)}{f\left(0.5\right).g\left(0.5\right)}\right\}$ traces, respectively. Table S1 (Supplementary File) categorizes the selected solid-state reaction mechanisms into four distinct models: reaction, diffusion, nucleation, and geometrical contraction models.

The activation energy and pre-exponential factor associated with the formation of activation complexes during the thermal degradation of banana peel were determined utilizing model-free isoconversional kinetic methods. The methodologies employed include the Starink (STK) regular integral [55] and the Friedman (FR) differential integral [56,57,58] models. These model-free approaches were selected based on recommendations from [59,60], which underscore the reliability of obtaining kinetic data through model-free methods as opposed to model-fitting techniques. Esmizadeh et al. [61] recognized that parameters derived from model-fitting can provide estimates of potential reaction mechanisms, in addition to kinetic and thermodynamic data at a singular heating rate. However, the International Confederation for Thermal Analysis and Calorimetry (ICTAC) [61] advocates for the application of multiple heating rates—specifically, a minimum of three—in model-free kinetic methodologies to ensure the reliability of kinetic data, thereby discouraging the use of a single heating rate. While several other regular integral model-free methods, such as the Ozawa-Flynn-Wall (OFW) [62,63] and Kissinger-Akahira-Sunose (KAS) [57,58,64], exist, the B-1.92 Starink (STK) model was selected due to its superior accuracy in approximating the temperature integral and its reduced sensitivity to noise [55]. Equations (S2) and (S3) delineate the mathematical formulations corresponding to the STK and FR model-free isoconversional methodologies, respectively. Furthermore, Equations (S4), (S5), (S6), and (S7) (Supplementary File) provide the mathematical expressions for estimating the entropy of activation (∆S), activation enthalpy (∆H), Gibbs free energy of the activated complex (∆G), respectively [4], and equilibrium constant (k).

### 5.2. Deconvolution of DTG Data

**Figure 6:**
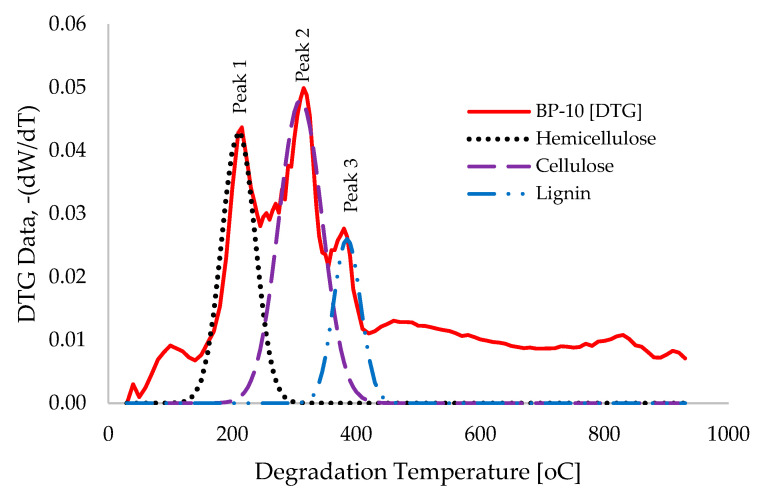
Graphical illustration of the “Original” and “Deconvoluted” DTG traces at 10 °C.min^−1^ of heating rate, depicting hemicellulose, cellulose, and lignin peaks.

The overlapping, multiple, and complex characteristics of the DTG traces for the thermal devolatilization of the BP biomass material, as presented in Figure 3b, suggest the independent contributions of the various components of lignocellulosic biomass [59,65,66]. Due to the intricate and multifaceted peaks associated with the pyrolysis of BP biomass, the direct application of the Criado master plot as indicated in Equation (S1) (Supplementary File) across the entire temperature range (30 to 950 °C) may yield inadequate theoretical predictions of this thermal behaviour [59]. Consequently, Gaussian mathematical functions (utilized in Matlab^TM^ 2018a) were employed to model and deconvolute the DTG traces for each of the principal components (hemicellulose, cellulose, and lignin), thereby facilitating the estimation of their respective reaction mechanisms. The selection of Gaussian mathematical functions for this analysis is attributable to their adaptability in adjusting the mean (centre) and standard deviation (width) to effectively fit a variety of real-world data [67]. Figure 6 illustrates the experimental and deconvoluted DTG traces for the pyrolysis of BP biomass at a heating rate of 10 °C.min^−1^, plotted against degradation temperature, highlighting significant peaks for hemicellulose, cellulose, and lignin occurring at degradation temperatures of 210 °C, 310 °C, and 385 °C, respectively, with peak widths of 27 °C, 35 °C, and 22 °C, respectively.

### 5.3. Criado Master Plots

**Figure 7:**
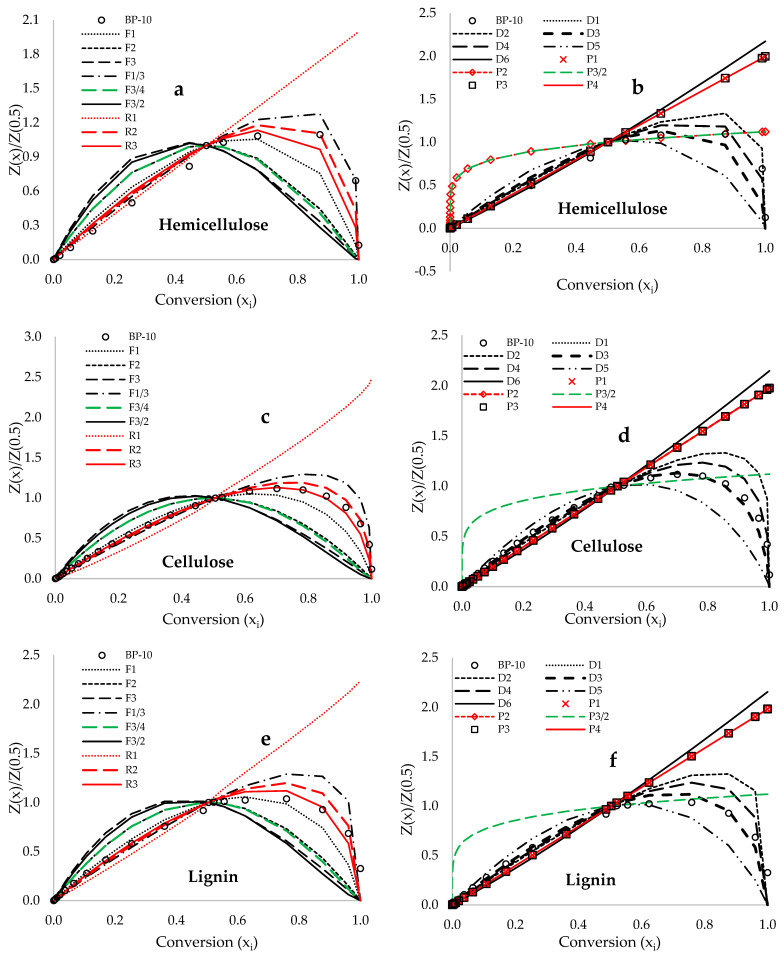
Criado’s master plots depicting both the reduced and deconvoluted experimental data Z(xi)/Z(0.5), obtained at a heating rate of 10 °C·min^−1^, alongside the theoretically estimated data plotted against conversion (xi). This analysis encompasses (**a**,**b**) hemicellulose, (**c**,**d**) cellulose, and (**e**,**f**) lignin decomposition, utilizing reaction-order models, diffusion models, nucleation models, and geometric contraction models.

Figure 7a–e presents the Criado master plots, which illustrate the relationship between reduced and deconvoluted experimental and theoretical estimations derived from the selected solid-state reaction models listed in Table S1 (Supplementary File), plotted against conversion (x_i_) for hemicellulose, cellulose, and lignin compositions. The plots in Figure 7 demonstrate the overlap of various reduced theoretical models with the reduced experimental data at different decomposition temperatures of the BP biomass compositions. For example, Figure 7a,b indicate that the 3D Diffusion model (Jander [D3]) represents the most accurate solid-state reaction mechanism for capturing the characteristic DTG traces associated with hemicellulose degradation. Figure 7c,d suggest that both the Contracting Sphere [R2] model and the 3D Diffusion model (Jander [D3]) yield accurate theoretical predictions for cellulose degradation. Furthermore, Figure 7e,f illustrates that the Contracting Cylinder [R3] model and the 3D Diffusion model (Jander [D3]) provide precise theoretical predictions for lignin decomposition. In summary, Figure 7a–e demonstrate that the 3D Diffusion model (Jander [D3]) offers the best fit for the decomposition of hemicellulose, cellulose, and lignin within BP biomass materials, followed by the R2 and R3 geometrical contraction models. Thus, the determination of the pre-exponential factor for the kinetics of this study was achieved using the 3D diffusion model (Jander [D3]).

### 5.4. Thermokinetic Data

**Figure 8:**
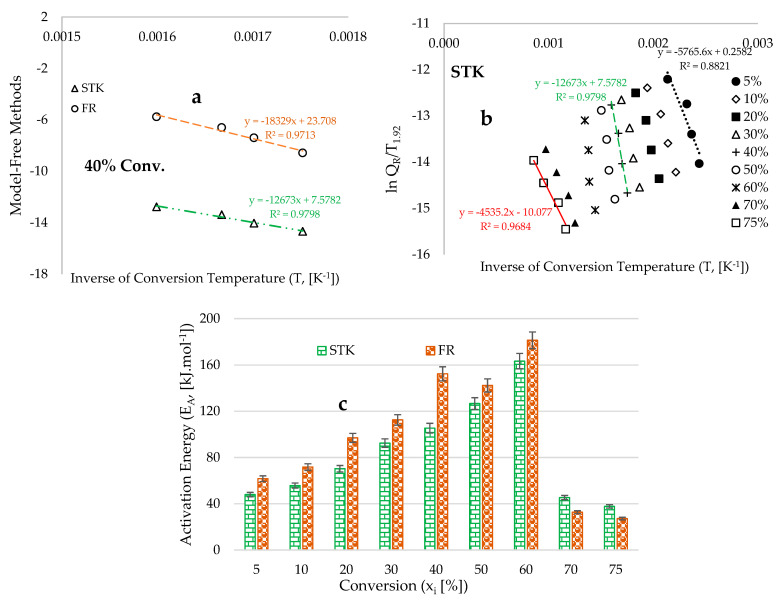
Graphs depicting (**a**) the estimated data derived from the chosen model-free kinetic methods as a function of the inverse of conversion temperature (T, [K^−1^]) at a constant conversion of 40%; (**b**) STK’s $\ln Q_{R}$ values plotted against the inverse of the conversion temperature (T, [K^−1^]) for various conversions ranging from 5% to 75%; and (**c**) the estimated activation energy obtained from the selected model-free methods plotted against conversions.

Figure 8a presents plots that depict estimated values derived from the application of selected model-free methods, plotted against the inverse of the conversion temperature [K^−1^] at 40% conversion. The slope and intercept of these inverse trends were fitted into the model-free kinetic methods (Equations (S2) and (S3)) to estimate the activation energy ($E_{A}$) and pre-exponential factor (A) across various conversions ranging from 5% to 75%. As illustrated in Figure 7a, the slope and intercept of the inverse trend obtained via the Friedman (FR) model-free kinetic method are the highest, indicating greater values for both the activation energy and pre-exponential factor. Figure 8b displays plots of estimated data derived from the Starink (STK) model-free method against the inverse of the conversion temperature for conversions ranging from 5% to 75%. The intercepts and slopes obtained from these plots reveal distinct trends at different degradation stages, with the highest values observed at the devolatilization stage, where the temperature spacing between the thermogravimetric analysis (TGA) traces is notably small. This observation suggests that the application of model-free methods for estimating kinetic parameters is significantly influenced by the temperature spacing between the TGA traces at varying heating rates; in essence, higher activation energy is associated with a reduced impact from changes in heating rate [47].

Figure 8c illustrates that the activation energy increases with the heating rate up to 60% conversion, which corresponds to the position of the maximum peaks observed in Figure 3b, resulting from the thermochemical conversion of cellulose and hemicellulose components of the fibre material. Beyond this 60% conversion threshold, a significant decrease in activation energy is noted in Figure 8c, which can be attributed to the pronounced effects induced by varying heating rates during the terminal stages of the TGA traces depicted in Figure 3a. Table 5 presents the thermokinetic data obtained for the formation of activated complexes from the reactants. The high coefficients of determination indicate a strong correlation between the experimental data and the selected model-free kinetic methods. Table 5 reveals that the overall activation energy obtained via the STK model is 82.8 ± 3.3 kJ.mol^−1^, which differs slightly from the estimate derived from the FR method (97.6 ± 3.9 kJ.mol^−1^). The FR method is classified as a differential isoconversional technique, and its practical application inevitably introduces some inaccuracies due to challenges in baseline determination and the effects of experimental noise, which may lead to significant errors [68]. Conversely, the STK iso-conversional model-free method is based on the regular integral approach that posits the independence of activation energy from conversion, thereby alleviating the limitations encountered by the Friedman (FR) differential isoconversional kinetic method [55,57]. Table 6 displays the activation energy data across the different pyrolysis conversion stages. Compared with the deconvoluted DTG data in Figure 6, the BP biomass’s moisture, hemicellulose, cellulose, and lignin composition have overall activation energies of 51.84 kJ.mol^−1^, 90.12 kJ.mol^−1^, 111.64 kJ.mol^−1^, and 95.70 kJ.mol^−1^, respectively.

Table 5 presents the overall activation enthalpies (ΔH) estimated using selected model-free isoconversional kinetic methods, which are positive, measuring 76.9 kJ.mol^−1^ for the STK model and 91.8 kJ.mol^−1^ for the FR model. These values indicate that the formation of activation complexes from reactants is an endothermic process [4]. Furthermore, the estimated overall entropy of activation for all selected model-free methods is negative and close to zero, suggesting that the formation of activation complexes is characteristic of a high molecularity reaction type, with a corresponding decrease in disorder. The computed values of Gibbs free energy for the formation of activation complexes (ΔG) are both positive for the two kinetic methods, implying that the activated complexes possess higher free energy than the reactants and that the process of their formation is non-spontaneous [4,28,69]. Additionally, the small positive values of the estimated regular equilibrium constant (k) for both selected kinetic models suggest that the Gibbs free energy values of the activated complexes (ΔG) are significantly positive (large), indicating a predominance of reactants over products (reactant-favoured) or a highly endergonic reaction [70].

Table 5 demonstrates that the difference between activation energy and activation enthalpy is quite small (that is $R.T_{M}=E_{A}-∆H\;\approx 5.9$ kJ.mol^−1^) and can be interchangeable to convey information regarding the energy difference between the ground state and the transition state [71] of the BP pyrolysis. The estimated activation energies for the pyrolysis of banana peel (BP) biomass, as determined by both STK and FR kinetic models, are significantly lower in comparison to those calculated for olive stones (124 kJ·mol^−1^) [72], corncobs (202.2 kJ·mol^−1^) [73], and cocoa shells (104.5 kJ·mol^−1^) [74]. Branca and di Blasi [75] reported banana peel activation energies of 82 and 86 kJ·mol^−1^ for devolatilization stages and 112 kJ·mol^−1^ for combustion. Using various kinetic models (Kissinger, KAS, FWO, Friedman, and CR), Kumar et al. [76] obtained values ranging from 108.42 to 201 kJ·mol^−1^. Similarly, Kumar et al. [13] estimated an average value of 107.42 kJ·mol^−1^ for raw peel using Arrhenius and CR models. This low activation energy estimated for the BP biomass pyrolysis, relative to other pure lignocellulosic materials, can be attributed to the catalytic effect of the naturally high concentration of inorganic mineral species, particularly potassium (15.93%) [75], as determined from the EDS data in Table 2. Furthermore, the total heat released during the thermal devolatilization of BP biomass can be quantified by estimating its gross calorific value (GCV), also known as high heating value (HHV), utilizing the theoretical relation (HHV[MJ/kg]=0.196(wt% of Fixed Carbon) + 14.119) as reported in [77]. Based on this relation, the estimated HHV value for pulverized BP biomass is 17.8 MJ/kg on a dry basis. In comparison, the estimated HHV values for corncob, *Areca catechu* fibre, and paper waste, as reported in [78], are 23.3 MJ/kg, 20.7 MJ/kg, and 14.5 MJ/kg, respectively. Additionally, available data on HHV for other biomass materials, such as rice husks (13.2 MJ/kg) and coffee husks (18.3 MJ/kg) [79], coconut shells (18.32 MJ/kg) [80], and tea waste and wood pellets (18.5 MJ/kg) [81], have also been corroborated.

The relatively low activation energy and activation enthalpy suggest that pulverized BP biomass is characterized as a softer biomass material [3]. This attribute results in decreased resistance to thermal decomposition, consequently accelerating reaction rates and enhancing the efficiency of material conversion into bioenergy. In summary, the low activation energy indicates high reactivity and facilitates the thermal breakdown of BP biomass, which corresponds with a diminished external energy requirement to sustain pyrolytic reactions. Furthermore, the high volatile matter content (67.8%) of BP biomass implies a substantial yield of bio-oil and syngas, as a significant proportion of organic materials is converted into volatile matter during thermal devolatilization [82]. Additionally, the elevated GCV of pulverized BP biomass, along with other advantageous characteristics such as low activation energy and reduced moisture content upon drying, positions BP biomass as a viable feedstock for pyrolysis and a promising alternative energy source. Moreover, treatments such as delignification and torrefaction may be employed on BP biomass to enhance its fuel properties, as well as to facilitate its utilization in biopolymer and biocomposite processing. This represents a recommendation for future studies by this research group.

## 6. Conclusions

This study presents a comprehensive assessment of the thermochemical conversion of pulverized banana peel (BP) utilizing thermogravimetric analysis (TGA) and derivative thermogravimetry (DTG) data, alongside a backpropagation deep learning methodology and thermokinetic analysis. Initial characterizations indicate that the BP material exhibits distinct properties typical of lignocellulosic fibrous materials, with moderate (6.5 wt%) and high volatile matter content (67.8 wt%), thereby positioning it as a suitable candidate for pyrolysis feedstock. The thermal degradation analysis of the BP biomass was performed using a TGA system across degradation temperatures ranging from 30 to 950 °C, under heating rates of 5, 10, 20, and 40 °C·min^−1^. This methodology elucidated a devolatilization temperature range of 190 to 660 °C for the pyrolysis of BP, characterized by multiple DTG peaks and a significant loss of material content.

The application of backpropagation deep learning techniques for the analysis of the resulting TGA traces facilitated the estimation of predicted TGA data that exhibited complete overlap with experimental scatter ($R^{2}\sim 0.99$), utilizing a total of 47,688 epochs. This level of modelling confidence was achieved through a series of modifications, including a gradual reduction in the learning rate from 5 to 1, as well as hyperparameter tuning, which culminated in the development of a DNN framework characterized by six hidden neurons distributed evenly across two hidden layers. The degradation temperature was identified as the most sensitive parameter influencing the thermochemical conversion of the BP biomass material.

The kinetic triplet for the formation of activated complexes was assessed for the process utilizing Criado master plots, along with model-free isoconversional kinetic methods, including the STK and FR approaches. By deconvolving the DTG traces obtained from the pyrolysis of BP biomass, the 3D Diffusion model (Jander [D3]) demonstrated the best fit for the decomposition of hemicellulose, cellulose, and lignin components within biomass materials, followed by the R2 and R3 geometrical contraction models. The overall activation energies estimated for both the STK and FR model-free isoconversional kinetic methods were 82.8 ± 3.3 kJ·mol^−1^ and 97.6 ± 3.9 kJ·mol^−1^, respectively. Additionally, the estimated thermodynamic parameters for the pyrolysis of BP indicated that the formation of activated complexes is endothermic, endergonic, and associated with lower disorder. In comparison to various biomass types documented in the literature, the thermal devolatilization of BP biomass materials presents a promising material for bioenergy generation.

In summary, the integration of inherently favourable reaction kinetics, characterized by low activation energy, with a highly reliable predictive tool—specifically, an accurate deep neural network (DNN) model—enhances the efficiency and cost-effectiveness of the banana peel pyrolysis process. This advancement positions the process as a compelling candidate for sustainable industrial waste-to-energy applications, facilitating the development of efficient bioreactors and the utilization of biomass materials in the degradation of polymers or the creation of sustainable biopolymers.

## Figures and Tables

**Table 1:** (**a**). Proximate analysis of the banana peel. (**b**). Ultimate analysis of the banana peel.

(**a**)				
**Moisture [%]**		**Fixed Carbon [%]**	**Volatile Matter [%]**	**Ash [%]**
6.5		18.7	67.8	7.0
(**b**)				
**C**	**H**	**N**	**S**	**O**
39.7	4.9	3.1	0.0	52.3

**Table 2:** EDS data for banana peel.

Element	Mass %	Atom %
C	39.69 ± 0.42	53.09 ± 0.59
O	37.22 ± 0.48	37.65 ± 1.00
Si	0.04 ± 0.01	0.02 ± 0.003
P	0.07 ± 0.01	0.03 ± 0.003
Cl	3.69 ± 0.15	1.81 ± 0.07
K	15.93 ± 0.37	7.12 ± 0.17
Au	3.35 ± 0.16	0.27 ± 0.09
**Total**	**100**	**100**

**Table 3:** Description of FTIR data.

Wavenumber [cm^−1^]	Description
3271	O-H stretching of alcohol present in cellulose/hemicellulose.
2848 and 2917	C-H stretching of alkanes.
1734	(C=O) _AC_ ester carbonyl stretching in pectin.
1577.0	C=C stretching of aromatic ring present in lignin.
1374	O-H bending of alcohol.
1026	C-O stretching of vibration.

**Table 4:** Statistical comparison of DNN and experimental data.

	EXPT	DNN
Mean Value [wt%]	50.99	51.01
Estimate Uncertainty (∆) [wt%]	1.37	1.36
Mean Bias Error [MBE]	0.02	
Coefficient of Determination ($R^{2}$)	0.9995	
True Error ($\varepsilon_{t}$) [%]	0.20	
Learning Rate ($L_{R}$)	5 → 1	
Initial Loss Function ($C_{o}$)	35.88	
Final Loss Function ($C_{f})$	0.07	
Total Training Time ($t_{T}$) [min]	82.34	
Training Epochs	47,688	

**Table 5:** Estimated average thermo-kinetic data.

	R^2^	E_A_	A	ΔS	ΔH	ΔG	k
STK	0.96	82.8 ± 3.3	3.0 × 10^−1^	−0.34	76.9	322.1	1.4 × 10^−24^
FR	0.97	97.6 ± 3.9	1.0 × 10^9^	−0.16	91.9	218.0	7.1 × 10^−17^

E_A_ is the activation energy [kJ.mol^−1^]; A is the pre-exponential factor [s^−1^], ΔS is the entropy of activation [kJ.mol^−1^.K^−1^], ΔH is the activation entropy [kJ.mol^−1^], and ΔG is the Gibbs free energy of the activated complex [kJ.mol^−1^].

**Table 6:** Activation energy data across the different pyrolysis conversion stages.

	STK		FR	
Conversion [%]	E_A_ [kJ.mol^−1^]	A [s^−1^]	E_A_ [kJ.mol^−1^]	A [s^−1^]
5	47.94	7.74 × 10^−8^	61.65	1.04 × 10^2^
10	55.74	4.45 × 10^−7^	71.71	2.02 × 10^3^
20	70.30	1.58 × 10^−5^	96.97	6.83 × 10^5^
30	92.46	7.37 × 10^−4^	112.55	4.39 × 10^6^
40	105.36	4.04 × 10^−3^	152.39	6.18 × 10^9^
50	126.73	8.14 × 10^−2^	142.34	1.06 × 10^8^
60	163.35	2.57	181.25	2.77 × 10^9^
70	45.35	6.56 × 10^−9^	32.63	2.46 × 10^−2^
75	37.71	1.87 × 10^−9^	27.18	1.25 × 10^−2^

## Data Availability

The original contributions presented in this study are included in the article/Supplementary Material. Further inquiries can be directed to the corresponding author.

## Supplementary Materials

The following supporting information can be downloaded at https://www.mdpi.com/article/10.3390/polym18010122/s1. The document outlines the thermokinetic equations and the solid-state reaction mechanisms chosen for the investigation.

## References

[1] Hsiao C.-J., Hu J.-L. (2024). Biomass and Circular Economy: Now and the Future. Biomass.

[2] Wang Y., Wu J.J. (2023). Thermochemical Conversion of Biomass: Potential Future Prospects. Renew. Sustain. Energy Rev..

[3] Dhifallah I., Saadi W., Souissi-Najar S. (2025). Kinetic, Thermodynamic, and Reaction Mechanism Study of Raw Phragmites australis pyrolysis using Thermogravimetric Analysis. Comptes Rendus. Chimie.

[4] Vyazovkin S. (2024). Misinterpretation of Thermodynamic Parameters Evaluated from Activation Energy and Preexponential Factor Determined in Thermal Analysis Experiments. Thermo.

[5] Nath B., Chen G., Bowtell L., Graham E. (2024). Thermal Decomposition of Wheat Straw Pellets in a Nitrogen Environment: Characterization using Thermogravimetric Analyzer. Case Stud. Therm. Eng..

[6] Otaru A.J., Albin Zaid Z.A.A. (2025). On the Thermal Degradation of Palm Frond and PLA 3251D Biopolymer: TGA/FTIR Experimentation, Thermo-Kinetics, and Machine Learning CDNN Analysis. Fuel.

[7] Vyazovkin S. (2021). Determining Preexponential Factor in Model-Free Kinetic Methods: How and Why. Molecules.

[8] Vlaev L.T., Georgieva V.G., Genieva S.D. (2007). Products and Kinetics of Non-Isothermal Decomposition of Vanadium (IV) Oxide Compounds. J. Therm. Anal. Calorim..

[9] Braga R.M., Melo D.M.A., Aquino F.M., Freitas J.C.O., Melo M.A.F., Barros J.M.F., Fotes M.S.B. (2014). Characterization and Comparative Study of Pyrolysis Kinetics of the Rice Husk and the Elephant Grass. J. Therm. Anal. Calorim..

[10] Bongomin O., Nzila C., Mwasiagi J.I., Maube O. (2024). Comprehensive Thermal Properties, Kinetic, and Thermodynamic Analyses of Biomass Wastes Pyrolysis via TGA and Coats-Redfern Methodologies. Energy Convers. Manag. X.

[11] Huang X., Cao J.-P., Zhao X.-Y., Wang J.-X., Fan X., Zhao Y.-P., Wei X.-Y. (2016). Pyrolysis Kinetics of Soybean Straw using Thermogravimetric Analysis. Fuel.

[12] Chua H.S., Shabuddin M.F.S., Lee K.M., Tat T.K., Bashir M.J.K. (2024). Sustainable Bio-Oil from Banana Peel Waste Biomass: Optimization Study and Effect of Thermal Drying. Glob. NEST J..

[13] Kumar S.A.P., Nagarajan R., Prasad K.M., Anand B., Murugaveh S. (2019). Thermogravimetric Study and Kinetics of Banana Peel Pyrolysis: A Comparison of “Model-Free” Methods. Biofuels.

[14] Ameha B., Nadew T.T., Tedla T.S., Getye B., Mengie D.A., Ayalneh S. (2024). The use of Banana Peel as a Low-Cost Adsorption Material for Removing Hexavalent Chromium from Tannery Wastewater: Optimization, Kinetic and Isotherm Study, and Regeneration Aspects. RSC Adv..

[15] Mishra S., Prabhakar B., Kharkar P.S., Pethe A.M. (2023). Banana Peel Waste: An Emerging Cellulosic Material to Extract Nanocrystalline Cellulose. ACS Omega.

[16] Potnuri RSuriapparao D.V., Rao C.R., Sridevi V., Kumar A. (2022). Effect of Dry Torrefaction Pretreatment of the Microwave-Assisted Catalytic Pyrolysis of Biomass using the Machine Learning Approach. Renew. Energy.

[17] Otaru A.J. (2025). Kinetics Study of the Thermal Decomposition of Date Seed Powder/HDPE Plastic Blends. Bioresour. Technol. Rep..

[18] Kartal F., Dalbudak Y., Özveren U. (2023). Prediction of Thermal Degradation of Biopolymers in Biomass under Pyrolysis Atmosphere by Means of Machine Learning. Renew. Energy.

[19] Oracle Saudi Arabia (2022). What Is Deep Learning. https://www.oracle.com/sa/artificial-intelligence/machine-learning/what-is-deep-learning/#:~:text=(0:23)-,Top%205%20Reasons%20to%20Use%20Deep%20Learning,retraining%20it%20with%20new%20data.

[20] Chen C., Xie W., Cai Z., Lu Y. (2023). Deep Learning for Cardiotocography Analysis: Challenges and Promising Advances. Advanced Intelligent Computing Technology and Applications.

[21] Betancur-Ancona D., Peraza-Mercado G., Moguel-Ordoñez Y., Fuertes-Blanco S. (2004). Physicochemical characterization of lima bean (*Phaseolus lunatus*) and Jack bean (*Canavalia ensiformis*) fibrous residues. Food Chem..

[22] Park S., Kim S.J., Oh K.C., Cho L., Jeon Y., Lee C., Kim D. (2022). Thermogravimetric Analysis-Based Proximate Analysis of Agro-Byproducts and Predictions of Calorific Value. Energy Rep..

[23] EMA 502 CHNS-O Elemental Analyzer. https://www.velp.com/public/file/EMA502Brochure-260178.pdf?srsltid=AfmBOooZaGZik1O474knEtluquHv7dPfg4brJWVkaWmYGieU85ZvMaym.

[24] Giurlani W., Berretti E., Innocenti M., Lavacchi A. (2019). Coating Thickness Determination Using X-ray Fluorescence Spectroscopy: Monte Carlo Simulations as an Alternative to the Use of Standards. Coatings.

[25] Kelly J.M., Bohman B., Bennett D., Taylor N.L. (2025). Rapid Dough Making Quality Analysis of Wheat Flour using Fourier Transform Infrared Spectroscopy and Chemometrics. Food Chem..

[26] InstaNANO FTIR Functional Group Database Table with Search. https://instanano.com/all/characterization/ftir/ftir-functional-group-search/.

[27] Sharma R., Sheth O.N., Gujrathi A.M. (2016). Kinetic Modeling and Simulation: Pyrolysis of Jatropha Residue De-Oiled Cake. Renew. Energy.

[28] Otaru A.J., Albin Zaid Z.A.A. (2025). Thermal Degradation of Palm Fronds/Polypropylene Bio-Composites: Thermo-Kinetics and Convolutional-Deep Neural Networks Techniques. Polymers.

[29] Vyazovkin S., Chrissafis K., di Lorenzo M.L., Koga N., Pijolat M., Roduit B., Sbirrazzuoli N., Suñol J.J. (2014). ICTAC Kinetics Committee Recommendations for Collecting Experimental Thermal Analysis Data for Kinetic Computations. Thermochim. Acta.

[30] Piazza V., da Silva Junior R.B., Frassoldati A., Lietti L., Chiaberge S., Gambaro C., Siviero A., Faravelli T., Beretta A. (2024). Detailed speciation of biomass pyrolysis products with a novel TGA-based methodology: The case-study of cellulose. J. Anal. Appl. Pyrolysis.

[31] Sukarni S. (2020). Thermogravimetric analysis of the combustion of marine microalgae Spirulina platensis and its blend with synthetic waste. Heliyon.

[32] Kabenge I., Omulo G., Banadda N., Seay J., Zziwa A., Kiggundu N. (2018). Characterization of Banana Peels Wastes as Potential Slow Pyrolysis Feedstock. J. Sustain. Dev..

[33] Pambudi N.A., Firdaus R.A., Ulfa D.K., Nanda I.R., Surhano Syivarulli R., Gandidi I.M., Sumbodo W. (2025). Optimization of Hydrothermal Processing for Banana Peel Waste: Enhancing Solid Fuel Characteristics as a Renewable Energy Source. Results Eng..

[34] Khamsaw P., Sommano S.R., Wongkaew M., Willats W.G.T., Bakshani C.R., Sirilun S., Sunanta P. (2024). Banana Peel (Musa ABB cv. Nam Wa Mali-Ong) as a Source of Value-Adding Components and the Functional Properties of its Bioactive Ingredients. Plants.

[35] Nair R.R., Schaate A., Klepzig L.F., Turcios A.E., Lecinski J., Shamsuyeva M., Endres H.-J., Pepenbrock J., Behrens P., Weichgrebe D. (2023). Physico-Chemical Characterization of Walnut Shell Biochar from Uncontrolled Pyrolysis in a Garden Oven and Surface Modification by Ex-Situ Chemical Magnetization. Clean Technol. Environ. Policy.

[36] Kamsonlian S., Suresh S., Balomajumder C., Chand S. (2011). Characterization of Banana and Orange Peels: Biosorption Mechanism. Int. J. Sci. Technol. Manag..

[37] Yan X., Cao Z., Murphy A., Ye Y., Wang X., Qao Y. (2023). FRDA: Fingerprint Region Based Data Augmentation using Explainable AI for FTIR Based Microplastics Classification. Sci. Total Environ..

[38] Memon J.R., Memon S.Q., Bhanger M.I., Memon G.Z., El-Turki A., Allen G.C. (2008). Characterization of Banana Peel by Scanning Electron Microscopy and FT-IR Spectroscopy and its use for Cadmium Removal. Colloids Surf. B Biointerfaces.

[39] Obele C.M., Ofoegbu S.U., Awuzie C.I. (2019). Extraction of Pectin from Ripe Plantain Peel, Fabrication and Characterization of Composite (Pectin/PVA/Glycerol) Films Produced from the Extracted Pectin. J. Miner. Mater. Charact. Eng..

[40] Ndung’u P.W., Mwithiga G., Onyari C.N., Muriithi G., Mukono S.T. (2020). Evaluating the Surface Functional Groups on Banana Leaf Petioles and the Resultant Biochar for Potential Absorbance. J. Mater. Environ. Sci..

[41] Afolabi F.O., Musonge P., Bakare B.F. (2021). Evaluation of Lead (II) Removal from Wastewater using Banana Peels: Optimization Study. Pol. J. Environ. Stud..

[42] Anniwaer A., Chaihad N., Zhang M., Wang C., Yu T., Kasai Y., Abudula A., Guan G. (2021). Hydrogen-Rich Gas Production from Steam Co-Gasification of Banana Peel with Agricultural Residues and woody biomass. Waste Manag..

[43] Cao W., Li J., Luo L., Zhang X. (2016). Release of Alkali Metals During Biomass Thermal Conversion. Arch. Ind. Biotechnol..

[44] Saddawi A., Jones J.M., Williams A. (2012). Influence of Alkali Metals on the Kinetics of the Thermal Decomposition of Biomass. Fuel Process. Technol..

[45] Kambo H.S., Dutta A. (2014). Strength, Storage, and Combustion Characteristics of Densified Lignocellulosic Biomass Produced via Torrefaction and Hydrothermal Carbonization. Appl. Energy.

[46] Crombie K., Mašek O., Sohi S.P., Brownsort P., Cross A. (2013). The effect of pyrolysis conditions on biochar stability as determined by three methods. GCB Bioenergy.

[47] Albin Zaid Z.A.A., Otaru A.J. (2025). Low-Heating-Rate Thermal Degradation of Date Seed Powder and HDPE Plastic: Machine Learning CDNN, MLRM, and Thermokinetic Analysis. Polymers.

[48] Otaru A.J., Alhulaybi Z.A., Owoseni T.A. (2023). On the Hydrodynamics of Macroporous Structures: Experimental, CFD and Artificial Neural Network Analysis. Chem. Eng. J. Adv..

[49] Sircar A., Yadav K., Rayavarapu K., Bist N., Oza H. (2021). Application of Machine Learning and Artificial Intelligence in Oil and Gas Industry. Pet. Res..

[50] Liquet B., Moka S., Nazarathy Y. (2021). The Mathematical Engineering of Deep Learning, Chapter 6. https://deeplearningmath.org/drafts/chap6.pdf.

[51] Mishra M. (2023). The Curse of Local Minima: How to Escape and Find the Global Minimum. https://mohitmishra786687.medium.com/the-curse-of-local-minima-how-to-escape-and-find-the-global-minimum-fdabceb2cd6a#:~:text=Use%20a%20different%20optimization%20algorithm%3A%20A%20different%20optimization%20algrithm%2C%20such,stuck%20in%20a%20local%20minimum.

[52] Kabir H., Garg N. (2023). Machine Learning Enabled Orthogonal Camera Goniometry for Accurate and Robust Contact Angle Measurements. Sci. Rep..

[53] Panneerselvam L. (2023). Activation Functions and their Derivatives—A Quick and Complete Guide (Deep Learning). https://www.analyticsvidhya.com/blog/2021/04/activation-functions-and-their-derivatives-a-quick-complete-guide/.

[54] Yash (2024). The Challenge of Vanishing/Exploding Gradients in Deep Neural Networks. https://www.analyticsvidhya.com/blog/2021/06/the-challenge-of-vanishing-exploding-gradients-in-deep-neural-networks/.

[55] Starink M.J. (2003). The Determination of Activation Energy from Linear Heating Rate Experiments: A Comparison of the Accuracy of Isoconversion Methods. Thermochim. Acta.

[56] Friedman H. (1964). Kinetics of Thermal Degradation of Char-forming Plastics from Thermogravimetry-Application to a Phenolic Resin. J. Polym. Sci. Part C Polym. Symp..

[57] Han Y. (2014). Theoretical Study of Thermal Analysis Kinetics, Theses and Dissertations, Mechanical Engineering, University of Kentucky. https://uknowledge.uky.edu/cgi/viewcontent.cgi?referer=&httpsredir=1&article=1036&context=me_etds.

[58] Otaru A.J. (2025). Circular Economy: Kinetic-Triplet, Thermodynamic, and Gradient Descent Optimisation Algorithm of Deep Learning Models for the Thermal Degradation of Walnut Shell. Case Stud. Therm. Eng..

[59] Vyazovkin S., Burnham A.K., Favergeon L., Koga N., Moukhina E., Pẻrez-Maqueda L.A., Sbirrazzuoli N. (2020). ICTAC Kinetic Committee Recommendations for Analysis of Multi-Step Kinetics. Thermochim. Acta.

[60] Vyazovkin S., Wight C.A. (1999). Model-Free and Model-Fitting Approaches to Kinetic Analysis of Isothermal and Non-Isothermal Data. Thermochim. Acta.

[61] Esmizadeh E., Tzoganakis C., Mekonnen T.H. (2020). Degradation behavior of polypropylene during reprocessing and its biocomposites: Thermal and oxidative degradation kinetics. Polymers.

[62] Ozawa T. (1965). A New Method of Analyzing Thermogravimetric Data. Bull. Chem. Soc. Jpn..

[63] Flynn J.H., Wall L.A. (1966). A Quick, Direct Method for the Determination of Activation Energy from Thermogravimetric Data. J. Polym. Sci. Part B Polym. Lett..

[64] Kissinger H.E. (1957). Reaction Kinetics in Differential Thermal Analysis. Anal. Chem..

[65] Kim H., Yu S., Kim M., Ryu C. (2022). Progressive Deconvolution of Biomass Thermogram to Derive Lignocellulosic Composition and Pyrolysis Kinetics for Parallel Reaction Model. Energy.

[66] Choudhary J., Kumar A., Alawa B., Chakma S. (2024). Optimization and Prediction of Thermodynamic Parameters in Co-Pyrolysis of Banana Peel and Waste Plastics using AIC Model and ANN Modeling. Energy Nexus.

[67] Liu K.Q., Zhang Z.C., Ostadhassan M. (2023). The Application of Gaussian Distribution Deconvolution Method to Separate the Overlapping Signals in the 2D NMR Map. Pet. Sci..

[68] Vyazovkin S. (2006). Thermal Analysis. Anal. Chem..

[69] Albin Zaid Z.A.A., Otaru A.J. (2025). Thermal Decomposition of Date Seed/Polypropylene Homopolymer: Machine Learning CDNN, Kinetics, and Thermodynamics. Polymers.

[70] Otaru A.J., Albin Zaid Z.A.A., Alkhaldi M.M., Albin Zaid S.M.A., AlShuaibi A. (2025). The Bioenergy Potential of Date Palm Branch/Waste Through Reaction Modeling, Thermokinetic Data, Machine Learning KNN Analysis, and Techno-Economic Assessments (TEA). Polymer.

[71] Atkins P., de Paua J. (2006). Physical Chemistry for the Life Sciences.

[72] Bedoui A., Souissi-Najar S., Ouederni A. (2016). Thermal Degradation of Tunisian Olive Stines using Thermogravimetric Analysis (TGA). Int. J. Innov. Appl. Sci..

[73] Okot D.K., Bilsborrow P.E., Phan A.N., Manning D.A.C. (2023). Kinetics of Maize Cob and Bean Straw Pyrolysis and Combustion. Heliyon.

[74] Mumbach G.D., Alves J.L.F., da Silva J.C.G., di Domenico M., de Sena R.F., Marangoni C., Machado R.A.F., Bolzan A. (2022). Pyrolysis of Cocoa Shell and its Bioenergy Potential: Evaluating the Kinetic Triplet, Thermodynamic Parameters, and Evolved Gas Analysis Using TGA-FTIR. Biomass Convers. Biorefinery.

[75] Branca C., di Blasi C. (2015). A Lumped Kinetic Model for Banana Peel Combustion. Thermochim. Acta.

[76] Kumar Shrivastava D., Prasad Chakraborty J. (2024). Estimation of Kinetic and Thermodynamic Parameters During Thermal Decomposition of Raw, Boiled, and Torrefied Banana Peel through Arrhenius and CR methods. Therm. Sci. Eng. Prog..

[77] Demirbaş A. (1997). Calculation of Higher Heating Values of Biomass Fuels. Fuel.

[78] Sihombing Hv Setyawan E.Y., Ambarita H. (2020). Comparison of Calorific Value of Corncobs, Areca Nut Fiber and Paper Waste as Alternative Fuel. AIP Conf. Proc..

[79] Mhilu C.F. (2014). Analysis of Energy Characteristics of Rice and Coffee Husks Blends. ISRN Chem. Eng..

[80] Hoque M.M., Bhattacharya S.C. (2001). Fuel Characteristics of Gasified Coconut Shell in a Fluidized and a Spouted Bed Reactor. Energy.

[81] Güler B. (2024). Biomass Valorization: Comparative Analysis of Tea Waste Pellets and Wood Pellets for Steam Generation and Emission Profiles. Sustain. Energy Technol. Assess..

[82] Zhang Z., Li Z., Yan H., Yan J., Zhang W., Wang Z., Lei Z., Ren S., Shui H. (2025). Effect of volatile matter and ash composition on the migration of organic sulfur during pyrolysis of high-sulfur coal. J. Anal. Appl. Pyrolysis.

