# Supplementary material for: Backpropagation DNN and Thermokinetic Analysis of the Thermal Devolatilization of Dried Pulverized Musa sapientum (Banana) Peel
Source: Polymers (Basel). 2025 Dec 31;18(1):122. doi: 10.3390/polym18010122 (PMC12787760; doi:10.3390/polym18010122)
Supplement: Supplementary file 1 [file polymers-18-00122-s001.zip › polymers-4084379-supplementary.pdf]

Criado master plots and solid-state reaction mechanisms

$$\frac{Z(x_i)}{Z(0.5)} = \frac{f(x_i).g(x_i)}{f(0.5).g(0.5)} = \left(\frac{T_x}{T_{0.5}}\right)^2 \frac{(dx/dt)_x}{(dx/dt)_{0.5}} \quad \text{(S1)}$$

where 0.5 denotes conversion at x = 0.5.

Table S1. Selected solid-state reaction mechanisms (Adapted from [58]).

**Reaction Mechanisms**	$\mathbf{g}(\boldsymbol{x})$	$\boldsymbol{f}(\boldsymbol{x})$
**Geometrical contraction models (GCM)**		
One-dimension [R1]	x_i	1
Contracting sphere [R2]	$1-(1-x_i)^{1/2}$	$(2).(1-x_i)^{1/2}$
Contracting cylinder [R3]	$1-(1-x_i)^{1/3}$	$(3).(1-x_i)^{2/3}$
**Reaction-order models (ROM)**		
First-order reaction model [F1] or Mampel model	$-\ln(1-x_i)$	$(1-x_i)$
Second-order reaction model [F2]	$(1-x_i)^{-1}-1$	$(1-x_i)^2$
Third-order reaction model [F3]	$[(1-x_i)^{-2}-1]/2$	$(1-x_i)^3$
One-third order [F1/3]	$1-(1-x_i)^{2/3}$	$(3/2).(1-x_i)^{1/3}$
Three-quarters order [F3/4]	$1-(1-x_i)^{1/4}$	$(4).(1-x_i)^{4/3}$
Three-halves order [F3/2]	$(1-x_i)^{-1/2}-1$	$(2).(1-x_i)^{4/2}$
**Power law models (PLM)**		
Power law [P1]	x_i	1
Power law [P3/2]	${x_i}^{2/3}$	$(2/3).{x_i}^{-1/2}$
Power law [P2]	${x_i}^{1/2}$	$2{x_i}^{1/2}$
Power law [P3]	${x_i}^{1/3}$	$3{x_i}^{2/3}$
Power law [P4]	${x_i}^{1/4}$	$4{x_i}^{3/4}$
**Diffusion models (DFM)**		
1D Diffusion model [D1]	${x_i}^2$	$1/(2x_i)$
2D Diffusion model [D2]	$[(1-x_i).\ln(1-x_i)]+x_i$	$[-\ln(1-x_i)]^{-1}$
3D Diffusion model (Jander [D3])	$[1-(1-x_i)^{1/3}]^2$	$(3/2).(1-x_i)^{2/3}/[(1-(1-x_i)^{1/3})]$
Ginstling-Brounshtein [D4]	$1-(2x_i/3)-(1-x_i)^{2/3}$	$(3/2)/[((1-x_i)^{-1/3}-1)]$
Zhuravlev, Lesokin, Tempelman [D5]	$[(1-x_i)^{-1/3}-1]^2$	$(3/2).(1-x_i)^{4/3}/[((1-x_i)^{-1/3}-1)]$
Anti-Jander [D6]	$[(1+x_i)^{1/3}-1]^2$	$(3/2).(1+x_i)^{2/3}/[((1+x_i)^{1/3}-1)]$

Isoconversional Model-Free Methods

Starink (STK): $$ln\,[Q_R/T^{1.92}] = ln\left(\frac{A.E_A}{R.(g(x_i))}\right) - \left(\frac{E_A}{R}\right).\frac{1}{T} \quad \text{(S2)}$$

Friedman Method (FR) $$ln\left[\frac{dx_i}{dt}\right] = ln\left[Q_R\,\frac{dx_i}{dT}\right] = -\left(\frac{E_A}{R}\right).\frac{1}{T} + lnA + lnf(x_i) \quad \text{(S3)}$$

Thermodynamic Parameters

Entropy of activation: $$\Delta S = R.\ln\left(\frac{A.h}{B_K.T_M}\right) \quad \text{(S4)}$$

Activation enthalpy: $$\Delta H = E_A - R.T_M \quad \text{(S5)}$$

Gibbs free energy of the activated complex: $$\Delta G = \Delta H - T_M.\Delta S \quad \text{(S6)}$$

Equilibrium constant: $$k = \exp\,(-\Delta G/(E_A - \Delta H)) \quad \text{(S7)}$$

where Q_R is the heating rate, T is temperature, h denotes Planck's constant (6.626 x 10^{-34} J.s^{-1}), B_K represents the Boltzmann constant (1.3806 x 10^{-23} J.K^{-1}) and T_M signifies the final or maximum temperature of constant conversion, measured in Kelvin.
